# Preliminary Clinical Outcomes of Human Umbilical Cord Mesenchymal Stem Cell‐Derived Exosomes in Chronic Rhinosinusitis With Nasal Polyps: A Case Report

**DOI:** 10.1111/jcmm.71166

**Published:** 2026-05-12

**Authors:** Manoochehr Avatef‐Fazeli, Gelavizh Rostaminasab, Iman Janghorban Esfahani, Amir Barimani, Kamran Mansouri

**Affiliations:** ^1^ Clinical Research Development Centre, Imam Khomeini, Mohammad Kermanshah and Farabi Hospitals Kermanshah University of Medical Sciences Kermanshah Iran; ^2^ Department of Biomedical Engineering, Graduate School Pukyong National University Busan South Korea; ^3^ Medical Biology Research Centre, Health Technology Institute Kermanshah University of Medical Sciences Kermanshah Iran

**Keywords:** chronic rhinosinusitis with nasal polyps, exosomes, mesenchymal stem cells, polyposis, regenerative medicine, rhinosinusitis

## Abstract

Chronic Rhinosinusitis with Nasal Polyps (CRSwNP) is a persistent inflammatory disease that may be resistant to medical management and recurs with traditional surgery. We report a 28‐year‐old male with a history of CRSwNP with comorbid asthma, who was resistant to all medical management, including saline irrigation, intranasal corticosteroids (INCS), and multiple courses of antibiotics, and elected against surgical intervention. Nasal endoscopy and imaging studies demonstrated bilateral polyposis and opacification of the ostia of all sinuses (Meltzer score 5 and Lund‐Mackay score of 18). Serum IgE level remained significantly high. The patient declined surgical intervention but consented to treatment with human umbilical cord MSC‐Exo (hUCMSC‐Exo) intramucosal and intrapolyp injections while continuing his standard regimen of saline irrigation and INCS. The treatment was well tolerated, with only reported mild and transient epistaxis. At follow‐up after two months, the patient experienced a rapid decrease in SNOT‐22 score (56–6). However, the symptomatic relief had abated, and the symptoms returned gradually but did not reach baseline (SNOT‐22 increased to 41). Conversely, asthma control remained stable during this time, and the serum IgE level showed an overall negative trend. Given the single‐patient, uncontrolled design with concurrent INCS and short follow‐up, causality cannot be inferred. This case supports feasibility/tolerability and generates hypotheses for larger controlled studies of this cell‐free approach.

**Abbreviations**
AR: Allergic Rhinitis
CRS: Chronic Rhinosinusitis
CRSwNP: Chronic Rhinosinusitis with Nasal Polyps
hUCMSC: Human Umbilical Cord Mesenchymal Stem Cell
INCS: Intranasal Corticosteroid
**LABA**
: Long‐acting β_2_‐agonist
**LMK**
: Lund–Mackay
MSC: Mesenchymal Stem Cell
MSC‐Exo: Mesenchymal Stem Cell Derived Exosomes
NPS/Meltzer: Nasal Polyp Score
**NTA**
: Nanoparticle tracking analysis
OCS: Oral Corticosteroid
**SABA**
: Short‐acting β_2_‐agonist
SNOT‐22: Sino‐Nasal Outcome Test
**TFF**
: Tangential‐flow filtration

## 1 Introduction

Chronic Rhinosinusitis with Nasal Polyps (CRSwNP), an inflammatory condition, is often characterised by nasal obstruction, congestion, anosmia, and facial pain. It affects 5%–13% of the population and causes an annual healthcare burden of $10–13 billion in the United States (1, 2). Type‐2 related inflammatory comorbidities like asthma and allergic rhinitis can make CRSwNP management problematic. Many of these patients become resistant to standard treatments of intranasal corticosteroids (INCS), saline irrigation, and surgery (3–5). For patients with CRSwNP, often driven by type‐2 inflammation and frequently accompanied by asthma, current guidelines support biologics in appropriately selected, steroid‐refractory cases. Randomised trials show that dupilumab (anti‐IL‐4Rα), mepolizumab (anti‐IL‐5), and omalizumab (anti‐IgE) reduce polyp burden and improve patient‐reported outcomes when added to INCS. However, access and cost barriers, heterogeneous responses, and relapse persist, underscoring the need for additional options [1]. Mesenchymal stem cell‐derived exosome (MSC‐Exo), as a new cell‐free therapy with anti‐inflammatory and immunomodulatory properties, offers a potential alternative (7–9). In preclinical models of allergic airway disease, MSC‐Exos have been shown to reduce type‐2 cytokine activity and eosinophil recruitment, promote anti‐inflammatory macrophagepolarisation, dampen dendritic‐cell activation, and support epithelial‐barrier repair (10–12). Likewise, mesenchymal stem cell therapy has demonstrated similar anti‐inflammatory effects in a **chronic rhinosinusitis mouse model** (13). Clinical evidence for intranasal MSC‐Exos in CRSwNP remains scarce. Therefore, we report a single‐patient experience to outline feasibility and tolerability and to generate hypotheses for future controlled studies.

## 2 Case Description

A 28‐year‐old man presented with a five‐year history of facial pain, anosmia, nasal obstruction, and purulent discharge. His past medical history was notable for asthma. Over the preceding two years, he had received intranasal corticosteroid/azelastine spray, saline irrigation, inhaled corticosteroids combined with long‐acting β_2_‐agonists, antihistamines, and recurrent antibiotic courses for acute exacerbations. Despite maximal medical therapy, he used a short‐acting β_2_‐agonist nearly five times weekly to manage asthma‐related dyspnea.

**FIGURE 1:**
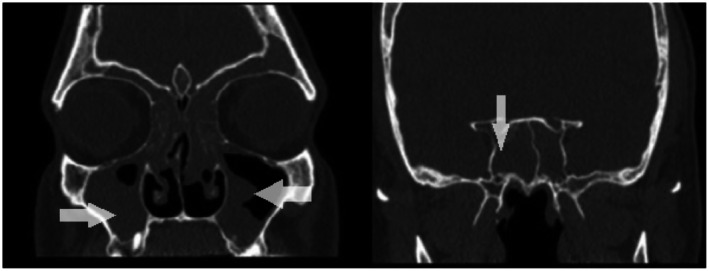
Coronal paranasal sinus CT scan, soft‐tissue window, Day 0. The image shows near‐total bilateral maxillary and complete bilateral sphenoid sinus involvement. Thick white arrows highlight the regions of opacification before treatment.

Objective assessment included nasal endoscopy, which revealed bilateral nasal polyps: Grade 3 on the left (extending below the inferior border of the middle turbinate) and grade 2 on the right (confined to the middle meatus), for a total Meltzer/NPS (Nasal Polyp Score) of 5 [2]. A paranasal sinus CT scan confirmed CRSwNP with a Lund–Mackay score of 18 (Figure 1). His Sino‐Nasal Outcome Test (SNOT‐22) score was 56, indicating substantial quality‐of‐life impairment. Routine laboratory studies (complete blood count, liver, and renal panels) were within normal limits, and total serum IgE was elevated at 376 IU/mL.

Because his symptoms remained refractory and he declined endoscopic sinus surgery, a multidisciplinary team comprising otolaryngologists, pharmacists, and stem cell researchers discussed different options. Biologic therapy (e.g., dupilumab, omalizumab) was discussed as guideline‐supported care for CRSwNP with asthma. Because of high cost and limited availability in our setting, and the patient's preference after shared decision‐making that included counselling on potential adverse effects and alternatives, biologics were deferred; instead, we proposed intranasal hUCMSC‐Exo as an add‐on to ongoing INCS and saline irrigation under ethics approval. The ethical approval was issued, and written informed consent was obtained from the patient for participation and for publication of anonymised clinical details and images. Human umbilical cord tissue (Wharton's jelly) from a single donor was used as the cellular source. Donor blood tested negative by PCR for HBV, HCV, HIV‐1/2, HTLV‐I/II, and CMV; VDRL and mycoplasma tests were also negative. Umbilical cord tissue was processed under sterile conditions, and human umbilical cord mesenchymal stromal cells (hUCMSCs) were isolated and expanded according to established procedures at the Medical Biology Research Centre and clean room under GMP protocol. Cells were cultured in DMEM supplemented with 100 U/mL penicillin and 100 μg/mL streptomycin at 37°C, 5% CO_2_, and characterised by flow cytometry: Positive for CD73, CD90, CD105, CD44 and negative for CD45, CD34, CD31, HLA‐DR. Karyotyping showed a normal chromosomal profile, and tri‐lineage differentiation (adipogenic, osteogenic, chondrogenic) was confirmed at passage 3. For exosome/small extracellular vesicle (sEV) production, hUCMSCs at passages 3–5 were grown to 70%–80% confluence, washed with PBS, and incubated for 48 h in serum‐free low‐glucose DMEM/F12. Conditioned medium was collected, and MSC‐Exos were isolated using tangential‐flow filtration (TFF). Vesicle morphology was confirmed by transmission electron microscopy, and identity was verified by Western blotting for canonical markers (CD63, CD9, CD81). Nanoparticle tracking analysis showed a modal particle diameter of 150–230 nm with a concentration of [4–4.5 × 10^9^] particles/mL. Endotoxin was not detected by a sensitive gel‐clot LAL assay, and aerobic/anaerobic bacterial and fungal cultures were negative. Preparations were aliquoted in 2 mL vials and stored at −80°C until use.

These prepared hUCMSC‐derived exosomes were administered via intramucosal and intrapolyp injections on Days 0, 7, 28, and 42. *Following the placement of a cotton swab soaked in lidocaine 10% and naphazoline 0.1% in each side of the nasal cavity for 10 min, intranasal injections were performed endoscopically using a* **27G** *needle at approximately* **2–3 mm** *depth into all polypoid tissue and the inferior turbinate head*.

Each session, the total dose of exosomes was 16–18 × 10^9^ particles in 4 mL, with 2 mL injected into each side of the nasal mucosa. Immediate tolerability was good; mild self‐limited epistaxis occurred post‐injection. The patient was observed for 30 min after each procedure and queried for adverse events at every visit. Additionally, he self‐administered twice daily topical MSC‐Exo spray from Days 17 through 26, with a total volume of 4 mL at the same dose. Treatment commenced in May 2023 and continued through late June 2023.

## 3 Follow‐Up and Outcomes

**FIGURE 2:**
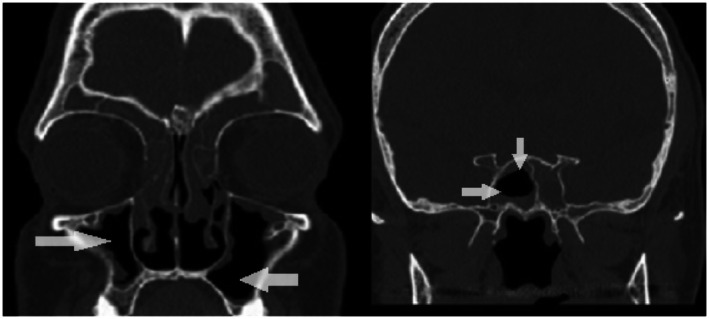
Coronal paranasal sinus CT scan, soft‐tissue window, Day 17. The image demonstrates a conspicuous reduction in maxillary sinus opacification and partial sphenoid improvement. White arrows and labels indicate areas of signal clearance corresponding to early radiologic response (Lund−Mackay score improved from 18 to 14).

Follow‐up assessments on Days 7 (pre‐second injection), 17 (Figure 2), 28 (pre‐third injection), 36, 42 (pre‐fourth injection), and 60 included SNOT‐22 scoring, endoscopic grading, and monitoring for adverse events (Table 1). Note that visits on days 17 and 36 were unscheduled (opportunistic).

**TABLE 1:** Summary of the patient's clinical course over seven visits.

Day	SNOT‐22	Meltzer scoring	Lund−mackay score	IgE (IU/mL)
0 (pre‐first injection)	56	5	18	376
7 (pre‐second injection)	24	4		
17	11	6	14	
28 (pre‐third injection)	6	6		
36	32	6		268
42 (pre‐fourth injection)	25	5		
60	41	5		

The SNOT‐22 decreased from 56 at baseline to **6** by Day 28 (Δ = −50), exceeding commonly cited MCIDs (≈9–12) for clinically meaningful change; however, the score subsequently rebounded to 41 by Day 60. Given the uncontrolled design and concurrent intranasal therapy, these fluctuations should be interpreted.

Asthma control improved progressively: He discontinued use of short‐acting β_2_‐agonists by Day 60. The only adverse event we noticed was mild epistaxis immediately following injections, which resolved spontaneously. Repeat laboratory tests at day 60 showed no evidence of organ dysfunction.

### 3.1 Patient Perspective

“I preferred to avoid systemic biologics or surgery due to costs, access, and concerns about adverse effects. The intranasal injections were tolerable with local anaesthesia. My nasal symptoms clearly improved in the first weeks, but some returned after about a month. I would consider repeat or alternative delivery if available.”

## 4 Discussion

In this single‐patient observation, intranasal MSC‐Exos were added to stable standard therapy (INCS and saline irrigation) and were associated with a rapid but transient symptom change: SNOT‐22 fell from 56 to 6 by Day 28 (Δ = −50) and then rebounded to 41 by Day 60. This early improvement exceeds commonly cited MCIDs for SNOT‐22 (≈9–12 points), but the subsequent relapse and the uncontrolled design with concurrent treatment mandate caution. Natural fluctuation and regression to the mean are well recognised in CRS; thus, symptom changes in an uncontrolled single case must be interpreted with caution. CT opacification improved from 18 to 14 (Lund–Mackay) by Day 17, while endoscopic Meltzer/NPS fluctuated within 4–6 thereafter.

When benchmarked against established standards, phase 3 SINUS‐24 and SINUS‐52 trials of Dupilumab plus INCS reported mean SNOT‐22 reductions of approximately 25 to 27 points, CT score improvements of around 5 points, and nasal polyp score reductions between 2.06 and 2.11 at week 24 [3]. In omalizumab (POLYP‐1/2), Week‐24 absolute changes were −1.08 vs +0.06 and −0.90 vs −0.31 for NPS, and −24.7 vs −8.6 and −21.6 vs −6.6 for SNOT‐22 (omalizumab vs placebo), and Lund–Mackay improved about 4 points in main group [4]. Interestingly, total IgE paradoxically increased due to formation of IgE–omalizumab complexes despite lower free IgE [5]. In contrast, intranasal corticosteroid monotherapy over 16 weeks typically yields ~19‐20‑point SNOT‑22 improvements with ~4.8‑point Lund–Mackay in 12 weeks and ~1‑point endoscopic score reductions [6, 7].

The Day‐60 rebound could reflect the waning of a short‐lived local exposure, together with natural CRS fluctuation. Potential mechanisms underlying this transient effect include rapid nasal mucociliary clearance (~10 min in adults), limiting exosome residence time and short systemic half‐lives of extracellular vesicles (< 1 h in preclinical models) [7, 8, 9]. Preclinical allergic rhinitis/asthma studies demonstrate that repeated intranasal MSC‐Exo dosing or retention‐enhancing formulations (e.g., hydrogels) are required for sustained type‐2 inflammation suppression. These pharmacokinetic constraints likely explain our early SNOT‐22 improvement (Δ = −50) followed by rebound despite four injections over 6 weeks. Systemically, extracellular vesicles show rapid clearance with circulating half‐lives on the order of minutes to < 1 h in animal models; mucosal surfaces add another constraint, as nasal mucociliary clearance in adults typically occurs on the scale of ~10 min, limiting residence time for topically applied biologics [7, 8, 9]. Preclinical AR/asthma studies indicate that repeated intranasal dosing of MSC‐derived sEVs can sustain symptom and cytokine improvements over days to weeks, and formulations designed to prolong local retention (e.g., hydrogels/encapsulation) are being explored to extend effect duration. These observations are consistent with a model in which frequent or depot‐style delivery is needed to maintain clinical benefit [10, 11, 12, 13]. Taken together, our case shows an early symptom signal with modest structural change (Meltzer 4–6; early LMK 18 → 14), contrasted with the controlled, durable NPS/CT improvements seen with guideline‐supported biologics over 24–52 weeks. The data here remain hypothesis‐generating: Future work should test standardised intranasal maps (sites/volumes/depths), prespecified NPS and LMK‐CT endpoints, biomarkers, and delivery strategies in controlled designs to establish safety, durability, and any role relative to biologics or surgery [3, 4, 14].

While our case suggested promising clinical and radiologic responses of intranasal MSC‐Exo, the exact positioning in the current CRS algorithm is unclear. Should it replace biologic agents? Or be used as an adjunct to the current standard treatment? Maybe it could serve as a temporary measure before surgery. Answering these questions requires further research.

This suggests that the current delivery method is suboptimal for this therapy [15]. Potential optimisation strategies should be considered to improve the local persistence and therapeutic efficacy of exosome delivery. These include formulation approaches such as hydrogel‐based or muco‐adhesive sustained‐release systems to prolong nasal mucosal residence time, increasing injection frequency to maintain steady local exposure, and evaluating alternative administration routes such as systemic or nebulised delivery for more uniform distribution. Future studies should compare these delivery strategies to determine the most efficient method for achieving durable clinical improvements in CRSwNP. Animal studies in mice, one of them in a colitis model and the other on the CRS model, demonstrated that intravenous MSC‐Exo and MSC (Mesenchymal Stem Cell) tend to migrate to inflamed areas while sparing normal mucosa. This suggests that systemic administration may work better for widespread inflammation [16, 17]. Another limitation is the absence of local inflammatory biomarker analyses, such as nasal lavage eosinophil counts and cytokine profiling (e.g., IL‐4, IFN‐γ). Including such markers in future studies would help clarify the immunomodulatory mechanisms and their relation to the observed clinical improvements and IgE trends. Additional limitations were the short 60‐day follow‐up, not considering long‐term effects and recurrence assessment. Because of cost constraints, we excluded eosinophil counts and Th2 cytokine measurements in nasal lavage fluid, thereby missing the opportunity to better characterize immunologic effects beyond serum IgE [14, 18], as well as histopathological evaluation of polyp tissue to confirm local histomorphologic and inflammatory changes [16, 19]. Similarly, the possibility of concurrent viral upper respiratory infections, a common exacerbating factor in chronic rhinosinusitis (CRS), was not documented and may have confounded symptom severity and inflammatory markers [20]. Future studies should include extended follow‐up (≥ 6 months) to assess durability, recurrence, and long‐term safety of intranasal exosome therapy.

## 5 Limitations

This is an uncontrolled, single‐patient observation with short follow‐up (60 days) and concurrent INCS/saline, precluding causal inference. The concurrent use of intranasal corticosteroids (INCS) and saline irrigation in this patient made it difficult to independently evaluate the specific therapeutic effect of the exosome intervention. The follow‐up period was limited to 60 days, which is insufficient to evaluate long‐term efficacy, safety, or recurrence. Additional follow‐up data (e.g., 6 months or 1 year) were not available because this study was restricted to an initial feasibility assessment under ethics approval. Some visits were opportunistic, and endpoints (NPS/LMK timing, biomarkers) were not fully pre‐specified; Day‐60 IgE was not obtained. Safety monitoring beyond immediate post‐procedure observation was limited, and no systemic adverse‐event surveillance was performed. Injection distribution was non‐uniform, likely favouring maxillary improvements on CT. We also did not measure local inflammatory biomarkers (eosinophil counts, IL‐4, IFN‐γ) in nasal lavage fluid, which limits mechanistic interpretation.

## 6 Conclusion

Intranasal MSC‐Exo administration appeared technically feasible and acutely tolerable in this patient, with an early but transient symptom signal and modest radiologic change under concurrent standard care. These observations do not establish efficacy or safety. Controlled studies with standardised delivery, schedules, and biomarker endpoints are required to determine any role relative to guideline‐supported biologics or surgery.

## Author Contributions

**Manoochehr Avatef‐Fazeli:** conceptualization, investigation, supervision, funding acquisition, writing – review and editing. **Kamran Mansouri:** conceptualization, methodology, investigation, validation, formal analysis, supervision, funding acquisition, project administration, writing – review and editing. **Gelavizh Rostaminasab:** methodology, data curation, formal analysis, validation. **Iman Janghorban Esfahani:** methodology, funding acquisition, resources, writing – review and editing. **Amir Barimani:** conceptualization, methodology, investigation, validation, formal analysis, writing – original draft, writing – review and editing.

## Funding

This work was supported by the Kermanshah University of Technology (1402507).

## Ethics Statement

This clinical study was approved by the Ethics Committee Board of Kermanshah University of Medical Sciences, Iran (Approval number: IR.KUMS.REC.1402.507; Date of approval: 2024‐01‐03). The title of the approved project is: Investigation of intramucosal injection of exosomes derived from umbilical cord mesenchymal stem cells on the treatment of chronic rhinosinusitis with polyposis—a case report.

## Consent

The patient provided written informed consent prior to participation. Umbilical cord donors provided informed consent for tissue donation and research use in accordance with institutional policies. The patient reviewed the case details and provided written informed consent for publication, including anonymised clinical images.

## Conflicts of Interest

The authors declare no conflicts of interest.

## Data Availability

The data that support the findings of this study are available on request from the corresponding author. The data are not publicly available due to privacy or ethical restrictions.
